# What will introducing and delivering new maternal vaccines cost in Ghana and Mozambique? A prospective analysis

**DOI:** 10.1016/j.vaccine.2025.126769

**Published:** 2025-03-07

**Authors:** Ranju Baral, Kwame Amponsa-Achiano, Iracema Barros, Patience Cofie, Patience Dapaah, Jessica A. Fleming, Chris Opoku Fofie, Sadaf Khan, Braiton Maculuve, Lauren Newhouse, Leonildo Nhampossa, Rosemond Owusu, Melanie Picolo, Diana Quelhas, Yara Voss De Lima, Clint Pecenka

**Affiliations:** a Center for Vaccine Innovation and Access, PATH, Seattle, United States of America; b Ghana Health Service, Accra, Ghana; c PATH, Maputo, Mozambique; d PATH, Accra, Ghana; e Ministry of Health, Maputo, Mozambique; f Independent consultant, Accra, Ghana; g Independent consultant, Maputo, Mozambique

**Keywords:** Maternal vaccines, Cost, Cost of delivery, Financial cost, Economic cost

## Abstract

**Introduction:**

The maternal vaccine landscape is expanding, including the anticipated global rollout of the now approved respiratory syncytial virus maternal vaccine. Integrating maternal immunization (MI) into health systems in low-and middle-income countries (LMICs) will require adaptation to existing immunization delivery platforms and have cost implications. In this study, we project the cost of maternal vaccine introduction and delivery in Ghana and Mozambique to help inform introduction decisions and ensure financial sustainability of future MI interventions.

**Methods:**

We used an activity-based prospective cost projection approach to estimate MI introduction and delivery costs for a five-year period from a health system perspective. Country stakeholders informed the strategies for MI delivery. Interviews with key immunization and maternal health program representatives informed on the anticipated health system adaptation requirements, activities, and resource needs taken into account in the cost projections. Supplementary data from a sample of sub-national health administrative units, vaccine stores, and health facilities also informed the costing analysis. Financial and economic costs are estimated and presented in 2023 USD units.

**Results:**

New maternal vaccines in both countries are anticipated to be delivered leveraging the existing maternal tetanus vaccine delivery practices. The non-vaccine cost of delivering one dose of maternal vaccine was estimated at $3.42 (financial) and $4.12 (economic) in Ghana, and $1.84 (financial) and $2.21 (economic) in Mozambique. Health worker training, communication and social mobilization, and program planning and coordination constitute the main cost drivers. Cost differences between countries are partly driven by the anticipated baseline coverage and baseline health system capacity gaps.

**Discussion:**

Very few studies exist on the costs of delivering maternal vaccine in LMICs. This study begins to fill this gap. These MI cost projections are comparable to other new vaccine introduction costs in similar settings, providing insights for local and global stakeholders seeking to understand costs of MI delivery.

## Highlights

• One of the few studies examining potential costs of maternal vaccination in LMICs.
• In-country experts and program representatives advised and validated key model inputs and assumptions.
• Maternal immunization introduction & delivery costs are comparable with other new vaccine introduction & delivery costs.

## 1 Introduction

Maternal immunization (MI) has emerged as a promising tool for addressing childhood infectious diseases in the earliest months of life. Vaccinating pregnant women boosts the level of maternal antibodies that can be transferred to the fetus through the placenta, conferring protection to infants in the critical first months after birth [1]. The maternal vaccine landscape is expanding. Several new maternal vaccines designed to protect young infants against illnesses are emerging, including respiratory syncytial virus (RSV) [2] maternal vaccine now licensed in some countries and a Group B *Streptococcus* (GBS) vaccine in development [3].

The licensed maternal RSV vaccine (Abrysvo, Pfizer) is in use in several high- and upper-middle-income countries [4]. Of relevance to low-and middle-income countries (LMICs), the World Health Organization (WHO) Strategic Advisory Group of Experts on Immunization (SAGE) made a global policy recommendation concerning RSV maternal vaccine in late 2024 [5]. A Gavi, the Vaccine Alliance (Gavi), final decision to support RSV interventions for LMICs is also anticipated in 2025. The prior Gavi commitment for RSV was contingent on the availability of a product that is licensed, WHO prequalified, and available at a reasonable price [6].

In many countries, vaccines provided to pregnant women (e.g., tetanus toxoid [TT]-containing vaccines), are delivered by leveraging the antenatal care (ANC) platform. The relatively small number of LMICs that have introduced maternal vaccines beyond TT highlights important implementation challenges. Nuances associated with novel maternal vaccines, such as administration during specific gestational age windows and seasonal delivery, will require adaptations to existing health systems (i.e., immunization and maternal health service delivery) and have cost implications. As global and country decision-makers explore mechanisms to deliver MI, cost of delivery is a critical factor for consideration. To date, few estimates of the delivery costs of vaccines for pregnant women in LMICs exist. No studies consider emerging RSV or GBS maternal vaccines specifically nor the potential need for an integrated delivery platform. The potential costs of establishing an MI delivery platform and the costs associated with recurrent delivery are also unknown.

In this study, we project the costs of establishing an MI platform and the recurrent costs of delivering MI in two African countries, Ghana and Mozambique. This study is conducted as a part of a multi-country costing study evaluating MI delivery costs across five countries in Asia and Africa. These cost projections will allow country decision-makers to better understand the cost implications of policy options and evaluate the economic feasibility of implementing MI interventions as products become available. These cost projections are also useful to global stakeholders shaping the further development of MI interventions and global health policy landscape. The data generated from this study can be used to inform further analyses such as cost-effectiveness and budget impact analyses.

## 2 Methods

#### 2.1.1 MI delivery strategy identification informed via stakeholder workshops

We assumed a pregnant woman's primary interaction with the health system as being through ANC services. Typically, globally maternal health programs manage ANC services, whereas, immunization programs manage vaccinations. As more maternal vaccines become available, the two programs need adaptations to create successful MI delivery platforms. Understanding country specific contexts, including specific opportunities and challenges pertaining to upcoming maternal vaccine introduction, is critical for informing robust MI cost projections. To gain insights into such contexts for this costing analysis, we conducted MI stakeholders' workshops in Ghana and Mozambique. During the workshops, 46 (Ghana) and 35 (Mozambique) national and regional ministry of health (MOH) representatives, Ghana Health Service (GHS) officials, academics, researchers, and non-governmental organization representatives deliberated on the anticipated adaptation needs for future MI interventions in their respective countries [7].

In both countries, stakeholders' deliberation concluded that future MI interventions will best be delivered by leveraging the existing mechanisms for delivering vaccines during pregnancy and that the maternal tetanus vaccine program provided proven pathways for implementing future MI. In Ghana, most women currently receive vaccines during pregnancy (TT-containing vaccines [Td]) from midwives during routine ANC visits. In Mozambique, however, most women receive vaccines at Expanded Programme on Immunization (EPI) clinics upon referral from the ANC visits. Guided by the workshop recommendations on MI delivery strategy, the study team conducted further data collection and analysis and estimated the costs of introducing and delivering maternal vaccines in Ghana and Mozambique.

#### 2.1.2 Analysis scope and perspective

We took a prospective cost projection approach to project MI delivery costs from respective health systems' perspectives. For each country, we projected costs for a five-year period for a nationwide introduction starting in 2024. All pregnant women were considered eligible to receive a single dose of maternal vaccine. The analysis considered costs incremental to the existing health system needed to support MI delivery via existing or adapted MI delivery platforms. Any direct expenses needed for introduction and recurring operations were considered financial costs to the government. While both countries are likely to receive external donor support for the new MI introduction in the future, such financing mechanisms were not considered for this analysis, except for the donated vaccine doses.

#### 2.1.3 Costing approach

We used an ingredients-based activity costing approach for cost projections. Activities needed to create a conducive platform for MI delivery in each country were first identified through mapping done by MOH representatives at national and sub-national levels, including focal points from immunization and maternal child health programs. Detailed activity maps were used to categorize specific activities into broader thematic areas aligned with the standard guidelines for estimating new vaccine introduction and delivery [[8], [9], [10], [11]]. Activities typically associated with vaccination programs such as program planning and management, procurement and distribution, training, demand generation, and service delivery were included. Within each activity category, the level and types of sub-activities varied by country, reflecting existing health system capacity gaps and resource needs. A detailed listing of sub-activities used to generate cost estimates are in Appendix Table 1 and 2.

We created an activity-resource map by identifying, quantifying, and mapping input resources, including labor, supplies, and other items needed to accomplish each sub-activity. Key MOH and service delivery agency representatives in each country validated the consolidated activity-resource map. We multiplied resource quantities by the corresponding unit costs to generate cost projections. The costing analysis followed standard guidelines for estimating the potential costs of new vaccine introduction and delivery [8], [9], [10], [11]. We conducted the analysis in Excel using a costing tool developed by PATH specifically for maternal vaccine costing analysis. The tool can be made available upon request.

Herein, we report two sets of cost estimates—financial and economic. Financial costs include direct financial outlays to the payer. Economic costs include financial costs plus the value of existing resource use, including the opportunity cost of existing staff time and donated goods. Vaccine doses were assumed to be donated to the country and, therefore, were only included in the economic cost estimates. Cost of vaccine procurement add-ons (e.g., shipping and handling costs) on vaccine doses and the cost of immunization supplies such as injection syringes, safety boxes were included as financial costs.

Costs were further categorized into introduction cost and recurrent costs. Introduction costs include costs associated with initial set-up such as introduction planning, training, social mobilization, and capital resource purchases such as cold chain equipment. Recurrent costs include costs of program operations on a routine basis, i.e., value that lasts less than a year, and include activities like procurement and distribution of vaccine supplies, vaccine delivery, routine monitoring and supervision, etc. Costs associated with systems adaptation, increased planning and coordination between the EPI and the maternal, newborn, and child health programs were considered both in introduction and recurrent costs. Costs of introduction (considered capital costs) were annualized and discounted at 3 % over a 5-year useful life for baseline estimates. Costs of capital item purchases, including cold chain equipment, were annualized over their respective estimated useful life years.

#### 2.1.4 Data collection

We collected data via key informant interviews, facility surveys, and secondary data review. Key informant interviews with the EPI and maternal health program leads as well as relevant focal points at the national level in each country informed detailed activities for costing, which were also supplemented by interviews with various sub-national health administrative unit representatives. We also leveraged immunization program's experience with recent new vaccine introductions to inform introduction related activities and unit costs.

Additionally, we collected data on service delivery at the point of care for existing services to supplement information gathered around MI service delivery resource requirements for future MI interventions. These data specifically informed potential recurrent costs of MI delivery. We used facility surveys for which a sample of health administrative units, vaccine stores, and health facilities, were purposively selected to represent geographic and programmatic diversity of the immunization program. The selection was done jointly with input from the respective national programs. In each country, data came from three subnational units (Ghana: Greater Accra Region, Bono East Region, and Upper West Region; Mozambique: Maputo Province, Zambezia Province, and Nampula Province). Two districts in each region/province were sampled. From each, two health facilities were selected for data collection (*N* = 12 health facilities in each country) (Appendix Table 3 and 4). Vaccine stores were also sampled at each level to inform costs of cold chain management, storage, and vaccine distribution.

Vaccine quantities and supplies used for cost projections were derived from projected key service recipient populations (i.e., pregnant women). We used the coverage of four or more ANC visits (ANC4+) by region for year 2022 to approximate maternal vaccine coverage instead of the TD maternal vaccine coverage as the RSV maternal vaccine is recommended to be given within a gestational age window around the third trimester.

Data collection occurred between June and October 2023 in Ghana and between October 2023 and March 2024 in Mozambique. Cost data were collected in local currency units (LCU) and are presented in LCU and United States dollar (USD) 2023 units.

#### 2.1.5 Product characteristics and vaccine cost

Neither maternal RSV nor GBS vaccines are yet available in most LMICs, so pricing remains largely unknown. Argentina (an upper middle-income country) is an exception [12]. For costing analysis, we assumed a lyophilized vaccine with a baseline vaccine price of US $3 per dose, which would be donated at no cost to countries. The cost of procurement add-ons was assumed to be paid by the government. In Ghana and Mozambique, UNICEF procures and delivers most vaccines and supplies to the national vaccine store (central vaccine store) based on an agreed shipment schedule.

#### 2.1.6 Shared inputs and capacity considerations

Costs of additional cold chain capacity requirements were calculated based on an assumed MI product (Table 1) and the anticipated dose quantity. Both countries have established vaccine distribution systems, which would need to be strengthened to accommodate increased volume due to MI and leveraged for the MI commodity distribution.

**Table 1:** Key data inputs and assumptions for maternal immunization cost-of-delivery analysis in Ghana and Mozambique.

Inputs	Ghana	Mozambique	Data sources
Target population and coverage			
Target population / Number of pregnant women	1,283,521	1,620,987	Ghana: District Health Information Management System (DHIMS), 2023, projection for 2022 Mozambique: Expanded Programme on Immunization (EPI) 2024, projections based on 2017 Census for year 2023
Population growth rate	1.5 %	2.5 %	Ghana: EPI Mozambique: EPI
Coverage rate for ANC4+ (Range across region)	88.00 % (75.7 %–94.8 %)	59.63 % (25.80 %–82.70 %)	Ghana: EPI, coverage for 2022 Mozambique: DHS 2022–2023
Product characteristics			
Presentation	3 doses per 2 ml vial, lyophilized		Assumption
Diluent	3 doses per 2 ml vial		
Vaccine packaged volume	9.3 cm3/dose		Assumed, based on RTS,S malaria vaccine
Doses in schedule	Single		Assumed, based on Target Product Profile
Cost of commodities			
Cost of vaccine per dose, USD	$3		Assumption
Cost per administration injection, USD	$0.06	$0.09	Ghana: Calculated, based on EPI expenses for 2022 Mozambique: Local supplier and UNICEF
Cost per reconstitution syringe, USD	$0.04	$0.09	
Cost per safety box (100 syringe capacity), USD	$0.50	$1.64	
Procurement add-on charges as a percentage of product cost:			
Freight, insurance, inspection	3.0 %	12.0 %	Ghana: Calculated, based on EPI expenses for 2022 Mozambique: Central de Medicamentos e Artigos Médicos (CMAM)/EPI
Handling fees	3.0 %	0.9 %	
Other taxes TOTAL	5.0 %	0.0 %	
TOTAL	11.0 %	12.9 %	
Service delivery			
Percentage of vaccination in routine (fixed) setting	80 %	95 %	Ghana: Assumed Mozambique: Ministry of Health
Percentage of vaccination in outreach setting	20 %	5 %	
Average time to vaccinate one person (range), in‑facility	5.05 min (2–12.5 min)	3.75 min (1–5 min)	Primary data collected during the health facility surveys
Average time to vaccinate one person (range), outreach	6.8 min (3.5–10 min)	3.75 min (1–5 min)	
Service delivery			
Useful life years for capital investments	10		Assumed [11]
Useful life years of introduction activities	5		
Discount rate	3 %		
Exchange rate [1 USD =]	11.9 GHS	62.3 MZN	Oanda, November 2023 [13]

To account for the incremental recurring costs of “operations,” the contributions to MI delivery were valued at 10 % (at baseline) of the total program cost based on direct allocation. These joint “operations” activities shared across the other programs included routine operations such as vaccine distribution, routine supervision, and monitoring. The overhead costs of operations are accounted for within each activity cost and consist of things like incremental labor, venue rental, and other supplies. The incremental time required to deliver maternal vaccine doses was estimated based on average vaccination time for TT-containing vaccine administration as reported by health workers during the health facility survey. For other shared resources, we assumed 100 % existing spare capacity in the existing health systems to accommodate MI interventions.

#### 2.1.7 Cost estimates

The total cost estimate is reported for a five-year period (2024–2028). The total cost is divided into introduction and recurrent cost. Estimates of unit costs including and excluding the commodity cost (vaccine and immunization supplies) are reported separately. Total introduction cost is divided by the total target population over the study period to generate introduction specific unit costs. The total recurrent costs over the study period are divided by the expected number of doses delivered to estimate the recurring unit cost per dose.

While the vaccine products are not yet available for LMICs, information on product characteristics and prices are assumed. The vaccine is assumed donated, so only included in the economic cost estimates. The cost of other immunization supplies, and the procurement add-ons cost of vaccine and immunization products are assumed to be paid by the government and are included in financial costs.

Baseline cost estimates are generated using the input values and assumptions noted in Table 1. A one-way sensitivity analysis was conducted to estimate the sensitivity of cost estimates with different input values for a subset of variables—for example, vaccine price, coverage, wastage rate, and service delivery time. The observed minimum and maximum values (based on health facility surveys) were used to generate a range of cost estimates. Results with alternative input values are presented in tornado graphs in Appendix Figure 1 and 2.

## 3 Results

### 3.1 Expected doses delivered and total costs

This analysis assumes all pregnant women to be eligible to receive a single dose of maternal vaccine. Annually, about 1.34 million and 1.74 million pregnant women were targeted for vaccination in Ghana and Mozambique, respectively. Assuming ANC4+ coverage as a proxy for MI coverage [see Table 1], roughly 1.18 million and 0.89 million pregnant women were expected to receive MI annually in Ghana and Mozambique, respectively [See Table 2].

**Table 2:** Target population, projected vaccinations, and projected total costs (USD), 2024–2028.

Metric	Ghana		Mozambique	
	All years	Annual average	All years	Annual average
Target population				
Total target population (pregnant women)	6,712,240	1,342,448	8,733,451	1,746,690
Total number of doses delivered	5,942,607	1,188,521	4,475,219	895,044
Total cost (in USD)				
Financial	28,348,971	5,669,794	15,533,534	3,106,707
Economic	51,435,438	10,287,088	32,645,198	6,529,040
Introduction cost, Financial	20,804,084	4,160,817	8,208,663	1,641,733
Introduction cost, Economic	22,345,530	4,469,106	9,006,865	1,801,373
Recurrent cost, Financial	7,544,887	1,508,977	7,324,871	1,464,974
Recurrent cost, Economic	29,089,908	5,817,982	23,638,333	4,727,667

At an assumed baseline vaccine price of $3 per dose, the total financial cost of the maternal vaccination program is projected to be $5.66 million and $3.10 million per year in Ghana and Mozambique, respectively. Similarly, the total annual economic cost is projected at $10.28 million in Ghana and $6.52 million in Mozambique. The total program cost includes the annualized introduction cost and the recurrent costs.

### 3.2 Unit cost estimates

The unit cost estimates across outcome metrics are given in Table 3. The estimated financial cost per eligible target population is $3.69 in Ghana and $1.66 in Mozambique. The economic cost per eligible target population is roughly double that of the financial cost estimate for both countries. Introduction cost per eligible target population is estimated at $2.56 (financial) and $3.09 (economic) in Ghana and $0.82 (financial) and $1.00 (economic) in Mozambique. The recurring financial cost of MI delivery is estimated at $1.27 and $1.64 in Ghana and Mozambique, respectively. Excluding the cost of vaccine and related immunization commodities, the financial cost of delivery per dose is estimated at $3.42 in Ghana and $1.84 in Mozambique (Table 3). Under alternative vaccine dose price assumptions ($1 and $5), the financial cost per dose administration in Ghana was $3.58 and $4.09 and that in Mozambique was $2.16 and $2.75. Similarly, the economic cost per dose administration, under alternative vaccine dose price assumptions ($1 and $5) was $5.43 and $10.54 in Ghana and $3.68 and $8.89 in Mozambique (see Appendix Figure 1 and 2 for additional scenarios).

**Table 3:** Unit cost estimates (in USD) of maternal immunization cost-of-delivery.

Metric	Ghana		Mozambique	
	Financial	Economic	Financial	Economic
Cost per eligible target population^1^	3.69	7.43	1.66	3.71
Introduction cost per eligible target population^2^	2.56	3.09	0.82	1.00
Recurrent cost per dose administered^3^	1.27	4.90	1.64	5.28
Cost of delivery per dose, with commodities^4^	3.83	7.99	2.46	6.29
Cost of delivery per dose, excluding commodities^5^	3.42	4.12	1.84	2.21

1 *Total cost over 5 years divided by total target population (expected number of pregnant women).*

2 *Total introduction cost (initial set-up cost) divided by total target population.*

3 *Total recurring costs divided by the total number of doses administered.*

4 *Total costs including immunization commodities: vaccine/injection supplies cost, divided by total number of doses administered.*

5 *Total cost excluding cost related to immunization commodities: vaccine/injection supplies cost, divided by total number of doses administered.*

### 3.3 Cost drivers

Introduction costs constitute roughly 70 % and 50 % of the total financial cost in Ghana and Mozambique, respectively. Of the total cost, procurement of immunization commodities constitutes 10 % and 19 % of financial cost, and 46 % and 56 % of economic cost in Ghana and Mozambique, respectively. Excluding procurement costs, health worker training for MI introduction is the major cost driver in both countries. Distribution of vaccines is the other major financial cost driver in Mozambique. Distribution of vaccines, demand creation activities, and cold chain expansion constitute other major cost drivers in Ghana (Table 4).

**Table 4:**
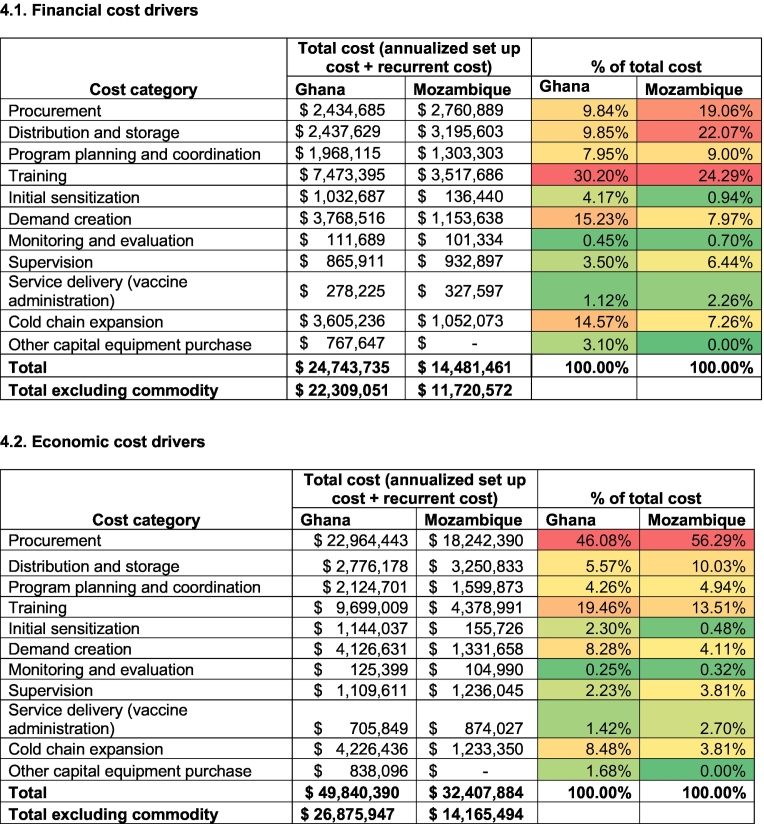
Drivers of total cost (annualized set up cost and recurrent cost), 2024–2028 (in USD).

## 4 Discussion

As new maternal vaccines requiring nuanced administration such as limited gestational age windows or seasonal delivery become available for use, effective delivery of such interventions will require adaptations to existing delivery platforms that have cost implications. Informed by in-country stakeholders (e.g., program managers and implementers), we identified activities and associated costs required for a successful MI introduction and delivery in two African countries: Ghana and Mozambique. Costs projected in this study are useful for country decision-makers to evaluate the economic feasibility of MI introduction in their respective settings. The value of this analysis extends beyond the country specific results presented here. It provides additional audiences with the ability to assess MI program costs and understand contextual factors that may inform future programs.

To summarize, the cost of maternal vaccine introduction and delivery is projected at $3.83 and $2.46 (financial), and $7.99 and $6.29 (economic) per dose, in Ghana and Mozambique, respectively. Excluding vaccine and related commodities, the unit cost of delivering a maternal vaccine is estimated at $3.42 and $1.84 (financial), and $4.12 and $2.21 (economic), in Ghana and Mozambique, respectively.

Comparison of findings between the two countries can be misleading and we urge readers to make comparisons cautiously. Country context such as the size of target population, expected vaccine coverage rates, current capacity gaps in the existing delivery systems and the anticipated activities needed for MI delivery all drive the differences in costs between countries. For example, while Ghana's cost per dose projections are higher than those of Mozambique, the target population (expected number of pregnant women) is higher in Mozambique compared to Ghana (annual average of 1.74 million versus 1.34 million), the anticipated number of vaccinations delivered is lower in Mozambique than in Ghana (annual average of 1.18 million versus 0.89 million) based on current ANC4+ coverage (Table 1). The expected maternal vaccine coverage in Mozambique is lower than in Ghana (average coverage 60 % versus 88 %. If Mozambique's anticipated coverage rate were to increase, this would further reduce the per dose delivery cost estimates making these estimates even lower than those from Ghana. The initial set-up cost/introduction per eligible target population is relatively higher in Ghana than in Mozambique, partly reflecting the larger denominator in Mozambique. In Ghana, introduction related activities constitute 70 % of total financial costs; initial sensitization and demand creation activities drives the introduction costs. In Mozambique, introduction costs constitute roughly half of the total financial costs.

Looking at the cost drivers, training of health workers along with orientation of related stakeholders and communities were identified as the major area needing initial investments for a successful MI introduction and delivery. This is reflected in the higher share of costs attributed to these activities in both countries. The economic cost captures the opportunity cost of the existing staff time to account for health worker engagement from immunization and the maternal health programs, unlike other typical vaccine introduction efforts focused on the immunization program alone. The engagement extends to all aspects of MI introduction and delivery, including introduction planning and coordination, training, communication and social mobilization, and other aspects of program operations. No substantial infrastructure adaptation was identified in either country outside of the strengthening of the cold chain capacity to accommodate the new vaccine storage capacity within the existing cold chain platforms. Cost of cold chain expansion and other capital expenditure required for MI introduction also contribute to higher unit costs in Ghana than in Mozambique. This is due to the larger number of districts and health facilities in Ghana compared to that in Mozambique (see Appendix Table 1 and 2).

Limited data on MI delivery cost projections are available in the literature. A cost projection of a seasonal maternal influenza immunization program in Malawi suggests the financial and economic costs per immunized pregnancy are $0.52 and $4.58, respectively [14] Under alternative scenarios, Pecenka et al. 2017 [14] estimates that the incremental delivery cost of a maternal influenza immunization program may be as low as some estimates of childhood vaccination programs, assuming the routine childhood immunization and antenatal care systems serve as the platform for an additional vaccination program. Stakeholders from both countries in our study also anticipated leveraging existing immunization platforms for future maternal vaccines, and findings in the current study also suggest that costs for MI introduction and delivery are generally comparable with other published vaccine delivery estimates (though our cost projections are somewhat higher). In Ghana, the non-vaccine cost of delivering a dose of RTS,S malaria vaccine was $2.30 (financial) and $4.09 (economic) [15]. Portnoy et al. projected a non-commodity cost of delivery for a range of countries with estimates of non-commodity delivery cost per dose of childhood vaccines of $3.04 ($0.90–$7.94) in Ghana and $1.17 ($0.39 - $2.78) in Mozambique [16]. Vaughan and colleagues report that the economic cost to deliver a single dose of vaccine in a health facility in LMICs ranges from $0.48 to $1.38 (2016 USD) [17]. The cost of introducing human papillomavirus vaccine via school- and health facility-based delivery ranges from $1.95 (financial) to $4.29 (economic) per dose [17]. While comparisons with other vaccine experiences should be made cautiously, our cost projections for MI are based on stakeholders' anticipated needs and current capacity gaps rather than a retrospective costing analysis.

This study has some limitations. Maternal vaccines considered in the current study are not yet available and, as such, necessitate making assumptions on vaccine product characteristics and cost, cold chain volume requirements, and coverage. To account for these uncertainties, we performed a one-way sensitivity analysis to evaluate the impact of such assumptions on the unit cost estimates. As expected, a lower product price and higher coverage of MI will reduce the unit cost projections for both countries and vice versa [see Appendix Figure 1 and 2]. The non-vaccine cost of delivery, which captures the operational cost of maternal vaccine delivery over a longer term, are robust and not highly influenced by the assumptions made around vaccine characteristics nor prices.

One strength of our study is the active participation of health program managers and focal persons, who informed data inputs. Adaptations to existing systems for creating conducive MI delivery are based on country baseline capacity and structures. The cost estimates generated in this study, therefore, reflect the resource needs in each country and account for existing gaps and needs therein at the time of study conduct. These anticipated resource needs may be different at the time of actual MI introduction decision-making depending on how far in the future that may occur. Evaluating the actual MI implementation costs from several settings once the programs are underway will be useful to assess the accuracy of our findings.

The RSV maternal vaccine was recently licensed and is being used in select high- and upper-middle-income countries [12], [18]. Accelerating access of this lifesaving maternal vaccine across LMICs is critical, as is preparing for future vaccines in the development pipeline such as for GBS. The costs of introducing new interventions are important information to help decision-makers evaluate the affordability and sustainability of intervention introduction in country. The cost estimates from this study provide useful insights to local and global stakeholders seeking to understand the costs of MI delivery, especially in LMICs where disease burdens are often highest.

## Funding

This study is based on research funded by the Bill & Melinda Gates Foundation. The findings and conclusions contained within are those of the authors and do not necessarily reflect positions or policies of the Bill & Melinda Gates Foundation. Under the grant conditions of the foundation, a Creative Commons license Attribution 4.0 Generic License has already been assigned to the Author Accepted Manuscript version that might arise from this submission.

## CRediT authorship contribution statement

**Ranju Baral:** Writing – review & editing, Writing – original draft, Validation, Supervision, Project administration, Methodology, Investigation, Formal analysis, Data curation. **Kwame Amponsa-Achiano:** Writing – review & editing, Validation, Supervision, Project administration. **Iracema Barros:** Writing – review & editing, Validation, Data curation. **Patience Cofie:** Writing – review & editing, Validation, Supervision. **Patience Dapaah:** Writing – review & editing, Validation, Investigation, Data curation. **Jessica A. Fleming:** Writing – review & editing, Methodology. **Chris Opoku Fofie:** Writing – review & editing, Validation, Supervision, Project administration. **Sadaf Khan:** Writing – review & editing, Methodology. **Braiton Maculuve:** Writing – review & editing, Validation, Supervision, Project administration. **Lauren Newhouse:** Writing – review & editing, Methodology. **Leonildo Nhampossa:** Writing – review & editing, Validation, Supervision, Project administration. **Rosemond Owusu:** Writing – review & editing, Validation, Data curation. **Melanie Picolo:** Writing – review & editing, Validation, Supervision, Project administration. **Diana Quelhas:** Writing – review & editing, Validation, Project administration, Data curation. **Yara Voss De Lima:** Writing – review & editing, Validation, Data curation. **Clint Pecenka:** Writing – review & editing, Supervision, Methodology, Conceptualization.

## Declaration of competing interest

The authors declare that they have no known competing financial interests or personal relationships that could have appeared to influence the work reported in this paper.

## Data Availability

Most data used in the paper are included in the paper. Additional information can be made available upon request.

## Appendix A Supplementary data

Supplementary material Image 1
