# Supplementary material for: What will introducing and delivering new maternal vaccines cost in Ghana and Mozambique? A prospective analysis
Source: Vaccine. 2025 Mar 7;49:None. doi: 10.1016/j.vaccine.2025.126769 (PMC11878280; doi:10.1016/j.vaccine.2025.126769)
Supplement: Supplementary file 1 — Supplementary material [file mmc1.docx]

#### **Supplementary materials**

#### Appendix Table 1: Detailed activities included in the costing analysis in Ghana

| **Activities** | **Frequency of activities** |
| --- | --- |
| **Vaccine and injection supplies procurement** |  |
| Procurement of maternal vaccine doses | Each year |
| Procurement of supplies- reconstitution syringe | Each year |
| Procurement of supplies- administration syringe | Each year |
| Procurement of supplies- safety boxes | Each year |
| **Distribution** |  |
| Monthly supply of vaccine and immunization supplies to the National store | Monthly delivered at the national store, shared cost |
| Distribution of vaccine and supplies from national to district stores | Quarterly (4 times) a year, shared cost |
| Quarterly immunization supplies collection trip by regional medical store to central medical store | Quarterly (4 times) a year, shared cost |
| Monthly collection of vaccines and immunization supplies by districts from regional stores | Monthly collection, shared cost |
| Monthly vaccine collection trips made by health facilities to district stores | Monthly, shared cost |
| Monthly operational costs of cold chain and maintenance at regional, district, and health facilities | Monthly, shared cost |
| **Program planning and coordination** |  |
| Set up technical working group (TWG) to help package information for National Immunization Technical Advisory Group (NITAG) decision- making | Three meetings in the introduction year only |
| Technical sub-committee meeting: planning and coordination | Five meetings in the introduction year only |
| Hold NITAG meeting to review policies and guidelines and recommend vaccine | Two meetings in the introduction year only |
| Interagency coordination meeting | Once in the introduction year |
| Introduction planning finalization workshop | Once in the introduction year |
| Consultative meeting with technical national level stakeholders | Once in the introduction year |
| Consultative meeting with technical national level stakeholders | Once in the introduction year |
| Annual microplanning at national, regional and district levels | Shared cost, each year |
| **Training** |  |
| Technical sub-committee meeting: training and surveillance | Five meetings in the introduction year only |
| Workshop to develop training package and tools | Once in the introduction year |
| Printing of training tools | Once in the introduction year |
| National level training of trainers (TOT) for regional officers | Once in the introduction year |
| TOT for district participants | Once in the introduction year |
| District level training | Once in the introduction year |
| Health facility level training for vaccinators | Once in the introduction year |
| **Initial sensitization** |  |
| Stakeholder mapping meeting | Once in the introduction year |
| Inception meeting between ministry of health representatives and regional directorate reps | Once in the introduction year |
| Stakeholder engagement and sensitization at regional level | Once in the introduction year |
| Stakeholder engagement and sensitization at district level | Once in the introduction year |
| Media sensitization and press briefing | Once in the introduction year |
| **Communication / demand creation** |  |
| Technical sub-committee meeting: social mobilization and communication (including radio message, social media message, TV message, etc.) | Five meetings in the introduction year only |
| Workshop for communication strategy development | Once in the introduction year |
| Workshop to develop communication materials | Once in the introduction year |
| Spokesperson training and sensitization | Once in the introduction year |
| Production and airing of radio and TV messages at national level | Once in the introduction year |
| Airing / broadcasting of radio messages at district level | Once in the introduction year |
| National launch event | Once in the introduction year |
| Regional launch events | Once in the introduction year |
| National TV coverage by spokesperson | Once in the introduction year |
| District radio station discussion | Once in the introduction year |
| Information sharing through Community Information Center (CIC) | Once in the introduction year |
| Orientation for champions (Queen mothers, celebrities, media person, etc.) | Once in the introduction year |
| Printing cost of communication materials | Once in the introduction year |
| **Monitoring and evaluation** |  |
| Monitoring and evaluation sub-committee meeting | Five meetings in the introduction year only |
| Modification of monitoring/reporting/recording tools | Once in the introduction year |
| Monitoring data review meeting with stakeholders | Once in the introduction year |
| Post introduction evaluation | Once in the introduction year |
| Printing of recording and monitoring tools | Each year. Shared cost |
| **Supervision** |  |
| National level supervision pre-introduction for readiness assessment | Once in the introduction year |
| National level supervision during launch | Once in the introduction year |
| District and facility supervision during introduction | Once in the introduction year |
| Routine quarterly supportive supervision by national, regional, district and facility officers | Each year, shared cost |
| **Service delivery** |  |
| Vaccination administration through routine Expanded Program on Immunization or antenatal care clinic (fixed strategy) | Each year, shared cost |
| Vaccination administration through routine outreach | Each year, shared cost |
| **Cold chain procurement** |  |
| Cold room walk in (capacity: 40 cu m) added at national level | In introduction year only |
| Refrigerator (capacity: 240 l) added to all regional vaccine stores | In introduction year only |
| Refrigerator (capacity: 240 l) added to all district vaccine stores | In introduction year only |
| One cold box (capacity: 18 l) and 1 vaccine carrier (capacity: 3 l) added to each health facility | In introduction year only |
| **Other capital equipment purchase** |  |
| Samsung tablets to support reporting and recording, 10 tables in each district | In introduction year only |

#### Appendix Table 2: Detailed activities included in the costing analysis in Mozambique

| **Activities** | **Frequency of activities** |
| --- | --- |
| **Vaccine and injection supplies procurement** |  |
| Procurement of maternal vaccine doses | Each year |
| Procurement of supplies- reconstitution syringe | Each year |
| Procurement of supplies- administration syringe | Each year |
| Procurement of supplies- safety boxes | Each year |
| **Distribution, storage and cold chain maintenance** |  |
| Quarterly receipt of vaccine and immunization supplies to the National store | Quarterly cost, shared cost |
| Quarterly distribution of vaccines from National (Central de Medicamentos e Artigos Médicos/ Expanded Programme on Immunization [EPI] stores) to the provincial stores | Quarterly (+2 trips added for additional ad hoc need) (outsourced to Prosolution), shared cost |
| Monthly distribution of vaccines from province to intermediary/district/health facility vaccine stores | Monthly (outsourced to Projecto Chegar), shared cost |
| Quarterly supervision of vaccine distribution process at all levels | Quarterly, shared cost |
| Operating cost of cold chain and maintenance at all levels | Annual, shared cost |
| **Program planning and coordination** |  |
| Hold Community of Immunization Experts (CoIE) meeting to review policies and guidelines and recommend vaccine. | Two meetings in the introduction year only |
| Interagency coordination committee meetings | Two meetings in the introduction year only |
| Vaccine introduction planning committee workshop | One workshop in the introduction year only |
| Planning and coordination sub-committee meetings for planning vaccine introduction | 12 meetings in the introduction year |
| Workshop to finalize the implementation plan | Once in the introduction year |
| National meeting for introduction inception | Once in the introduction year |
| Microplanning for maternal immunization (MI) introduction at province level | Once in the introduction year |
| Microplanning activity at district level | Once in the introduction year |
| Routine annual microplanning at province and district levels | Each year, shared cost |
| **Training** |  |
| Technical sub-committee meetings to plan and prepare for new vaccine introduction training | 12 meetings in the introduction year only |
| Workshop to develop training materials | Once in the introduction year |
| Printing of training materials | Once in the introduction year |
| Training of national level staff/trainers | Once in the introduction year |
| Training of trainers at the national level for province level staff | Once in the introduction year |
| Training of trainers at provincial level for district level staff | Once in the introduction year |
| Training of health workers | Once in the introduction year |
| **Initial sensitization** |  |
| [Orientation of professional organizations and societies on MI introduction](file:///C:\Users\rbaral\Box\MI%20Support\MI%20Support-INT\MI%20Support-INT-PAI\Activity%204\COD%20MODELS\Tables%20for%20manuscript_MOZAMBIQUE.xlsx#RANGE!C4) | Once in the introduction year |
| [Orientation of other ministries, NGOs and partners](file:///C:\Users\rbaral\Box\MI%20Support\MI%20Support-INT\MI%20Support-INT-PAI\Activity%204\COD%20MODELS\Tables%20for%20manuscript_MOZAMBIQUE.xlsx#RANGE!C39) | Once in the introduction year |
| [Orientation of other media at the National level](file:///C:\Users\rbaral\Box\MI%20Support\MI%20Support-INT\MI%20Support-INT-PAI\Activity%204\COD%20MODELS\Tables%20for%20manuscript_MOZAMBIQUE.xlsx#RANGE!C74) | Once in the introduction year |
| Orientation to traditional and religious group representatives, opinion leaders | Once in the introduction year |
| Province level stakeholder orientation on MI | Once in the introduction year |
| District level stakeholder orientation on MI | Once in the introduction year |
| **Communication / demand creation** |  |
| Communications technical sub-committee meeting | 12 meetings in the introduction year only |
| Workshop to develop communication materials | Once in the introduction year |
| Printing of communication materials and distribution costs | Once in the introduction year |
| Production/conception and airing of radio spots, TV spots, and various printed materials in local languages. | Once in the introduction year |
| Launch of MI vaccination program- national level | Once in the introduction year |
| Launch of MI vaccination program-province level | Once in the introduction year |
| Launch of MI vaccination program-district level | Once in the introduction year |
| Social mobilization through mobile health unit | Once in the introduction year |
| Community health actors orientation/training on MI vaccination | Once in the introduction year |
| **Monitoring and evaluation** |  |
| [Technical sub-committee meetings to update and finalize the monitoring and evaluation (M&E) tools for new vaccine introduction](file:///C:\Users\rbaral\Box\MI%20Support\MI%20Support-INT\MI%20Support-INT-PAI\Activity%204\COD%20MODELS\Tables%20for%20manuscript_MOZAMBIQUE.xlsx#RANGE!C4) | Six meetings in the introduction year only |
| [Workshop to update and finalize M&E tools](file:///C:\Users\rbaral\Box\MI%20Support\MI%20Support-INT\MI%20Support-INT-PAI\Activity%204\COD%20MODELS\Tables%20for%20manuscript_MOZAMBIQUE.xlsx#RANGE!C39) | Once in the introduction year |
| [Printing of monitoring tools and materials](file:///C:\Users\rbaral\Box\MI%20Support\MI%20Support-INT\MI%20Support-INT-PAI\Activity%204\COD%20MODELS\Tables%20for%20manuscript_MOZAMBIQUE.xlsx#RANGE!C109) | Once in the introduction year |
| Post-introduction evaluation (PIE) | Once in the introduction year |
| **Supervision** |  |
| Post/introduction supervision from national to province level | Once in the introduction year |
| Post/introduction supervision of districts from the province level | Once in the introduction year |
| Post introduction supervision at health facilities from districts | Once in the introduction year |
| Routine biannual (twice in a year) national to province level supervision | Two times each year, shared cost |
| Routine quarterly supervision of districts from the province level | Four times each year, shared cost |
| Routine monthly supervision at health facilities from districts | Twelve times each year, shared cost |
| **Service delivery** |  |
| Vaccination administration through routine EPI (fixed site) | Each year, shared cost |
| Vaccination administration through routine outreach/mobile brigade | Each year, shared cost |
| **Cold chain procurement** |  |
| Cold room walk in (capacity: 40 cu m) added at national level | In introduction year only |
| Refrigerator (capacity: 240 l) added to all district vaccine stores | In introduction year only |
| Refrigerator (capacity: 240 l) added to all province vaccine stores | In introduction year only |
| One cold box (capacity: 18 l) and 1 vaccine carrier (capacity: 3 l) added to each health facility | In introduction year only |
| **Waste management** |  |
| None |  |

#### Appendix Table 3: List of areas and facilities surveyed for costing in Ghana

**Key informant interviews**

| **Level** |  |
| --- | --- |
| National level | National Expanded Programme on Immunization, Ghana Health Service (GHS) |
|  | National Maternal and Child Health Program, Family Health Division, GHS |
|  | National Cold Room |
| Regional level | Greater Accra Regional Health Directorate |
|  | Bono East Regional Health Directorate |
|  | Upper West Regional Health Directorate |

**Health facility/vaccine store surveys**

**Vaccine stores**

| **Level** |  | **Facility name** | **Facility setting/type/level** |
| --- | --- | --- | --- |
| National level |  | Expanded Programme on Immunization/Vaccine Cold Room | National |
| Greater Accra |  | Greater Accra Health Directorate | Regional Vaccine store |
| Bono East |  | Bono East Regional Health Directorate | Regional Vaccine store |
| Upper West |  | Regional vaccine store | Regional Vaccine store |
| Bono East | Techiman Municipal | District vaccine store | District vaccine store |
| Bono East | Pru East | District vaccine store | District vaccine store |
| Greater Accra | Ada East | Ada East Health Directorate | District vaccine store |
| Greater Accra | GA West Municipal | Municipal Health Directorate | District vaccine store |
| Upper West | Daffiama Bussie Issa district | Daffiama Bussie Issa district | District vaccine store |
| Upper West | Sissala West | District vaccine store | District vaccine store |

**Health facilities**

| **Region name** | **District name** | **Facility name** | **Facility setting/type/level** |
| --- | --- | --- | --- |
| Greater Accra | Ada East | Ada Health Center | Health Center |
| Greater Accra | Ada East | Fantevikope Community Health Planning and Services (CHPS) | CHPS |
| Greater Accra | GA West Municipal | Achiaman Health Center | Health center |
| Greater Accra | GA West Municipal | Nsakina CHPS | CHPS |
| Bono East | Techiman Municipality | Fiaso CHPS | CHPS |
| Bono East | Techiman Municipal | Nsuta Health Center | Health Center |
| Bono East | Pru East | Parambo Health Center | Health Center |
| Bono East | Pru East | Kobre CHPS | CHPS |
| Upper West | Daffiama Bussie Issa | Fian Health Center | Health Center |
| Upper West | Daffiama Bussie Issa | Tuori and Worgberi CHPS | CHPS |
| Upper West | Sissala West | Duwie CHPS | CHPS |
| Upper West | Sissala West | Jeffisi Health Center | Health Center |

#### Appendix Table 4: List of areas and facilities surveyed for costing in Mozambique

**Key informant interviews**

| **Level** |  |
| --- | --- |
| National level | National Expanded Program on Immunization (EPI), Ministry of Health (MOH) Mozambique |
|  | National Maternal and Child Health Program (MCH), MOH Mozambique |
|  | National Medicines and Medical Supplies Store/ Central de Medicamentos e Artigos Médicos |
| Provincial level | Maputo Province EPI, MCH divisions and vaccine stores |
|  | Zambézia Province EPI, MCH and vaccine stores |
|  | Nampula Province EPI, MCH and vaccine stores |

**Health facilities**

| **Province name** | **District name** | **Facility name** | **Facility setting/type/level** |
| --- | --- | --- | --- |
| Maputo | Matola | Centro de Saúde Boquisso | Health Center |
| Maputo | Matola | Centro de Saúde Matola II | Health Center |
| Maputo | Magude | Centro de Saúde Magude | Health Center |
| Maputo | Magude | Centro de Saúde Motaze | Health Center |
| Zambézia | Quelimane | Centro de Saúde 24 de Julho | Health Center |
| Zambézia | Quelimane | Centro de Saúde Maquivale Sede | Health Center |
| Zambézia | Namacurra | Centro de Saúde Namacurra Sede | Health Center |
| Zambézia | Namacurra | Centro de Saúde Muceleiua | Health Center |
| Nampula | Nampula | Centro de Saúde Muhala Expansão | Health Center |
| Nampula | Nampula | Centro de Saúde Anchilo | Health Center |
| Nampula | Monapo | Centro de Saúde Monapo Sede | Health Center |
| Nampula | Monapo | Centro de Saúde Murruto | Health Center |

#### Appendix Figure 1: One way sensitivity of unit cost estimates for MI introduction and delivery in Ghana

**Panel 1.a: Cost per dose administration, financial**

**Panel 1.b: Cost per dose administration, economic**

**Panel 1.c: Cost of delivery per dose (excluding commodity cost), economic**

Appendix Figure 2: One way sensitivity of unit cost estimates for MI introduction and delivery in Ghana

**Panel 2.a: Cost per dose administration, financial**

**Panel 2.b: Cost per dose administration, economic**

**Panel 2.c: Cost of delivery per dose (excluding commodity cost), economic**
